# ADAMTS-12: Functions and Challenges for a Complex Metalloprotease

**DOI:** 10.3389/fmolb.2021.686763

**Published:** 2021-04-30

**Authors:** Yamina Mohamedi, Tania Fontanil, Santiago Cal, Teresa Cobo, Álvaro J. Obaya

**Affiliations:** ^1^ Departamento de Bioquímica y Biología Molecular, Universidad de Oviedo, Oviedo, Spain; ^2^ Departamento de Investigación, Instituto Ordóñez, Oviedo, Spain; ^3^ Instituto Universitario de Oncología, IUOPA, Universidad de Oviedo, Oviedo, Spain; ^4^ Departamento de Cirugía y Especialidades Médico-Quirúrgicas, Universidad de Oviedo, Oviedo, Spain; ^5^ Instituto Asturiano de Odontología, Oviedo, Spain; ^6^ Departamento de Biología Funcional, Área de Fisiología, Universidad de Oviedo, Oviedo, Spain

**Keywords:** ADAMTS-12, matrix metalloprotease, inflammation, arthritis, schizophrenia, cancer

## Abstract

Nineteen members of the ADAMTS family of secreted zinc metalloproteinases are present in the human degradome. A wide range of different functions are being attributed to these enzymes and the number of their known substrates is considerably increasing in recent years. ADAMTSs can participate in processes such as fertility, inflammation, arthritis, neuronal and behavioral disorders, as well as cancer. Since its first annotation in 2001, ADAMTS-12 has been described to participate in different processes displayed by members of this family of proteinases. In this sense, ADAMTS-12 performs essential roles in modulation and recovery from inflammatory processes such as colitis, endotoxic sepsis and pancreatitis. ADAMTS-12 has also been involved in cancer development acting either as a tumor suppressor or as a pro-tumoral agent. Furthermore, participation of ADAMTS-12 in arthritis or in neuronal disorders has also been suggested through degradation of components of the extracellular matrix. In addition, ADAMTS-12 proteinase activity can also be modified by interaction with other proteins and thus, can be an alternative way of modulating ADAMTS-12 functions. In this review we revised the most relevant findings about ADAMTS-12 function on the 20th anniversary of its identification.

## Introduction

Since its first annotation in the human genome 20 years ago (NCBI Gene ID: 81792), ADAMTS-12 has been associated to different biological processes in both physiological and pathological conditions, including development, cancer, osteoarthritis, neurological disorders and inflammation. Following ADAMTS-12 initial identification and characterization, eight more members of the ADAMTS (*ADisintegrin And Metalloprotease with ThromboSpondin domains*) family of proteinases have been identified, making a total of nineteen the number of members of this family of metalloproteases in mammals (Kelwick et al., 2015). In general, all the members share a complex multidomain architecture. This organization includes a signal peptide, a prodomain, a catalytic domain (proteinase domain characterized by an aspartic residue at the end of the consensus Zn^2+^-chelating motif HExxHxxGxxHD), a disintegrin-like domain, a central thrombospondin like type-1 (TSP-1) motif, a cysteine-rich domain, a spacer region and a variable number of TSP-1 repeats. Most of the members differ in the number of TSP-1 repeats at the carboxy-end region and also by the presence of additional motifs like PLAC, MuPr, *cub* or GON-1 domains (Porter et al., 2005; Kelwick et al., 2015).

Human ADAMTS12 gene maps at position 5p13 and spans approximately 368.45 Kb of genomic DNA containing a total of 24 exons (HGNC: 14605). Conceptual translation of human ADAMTS12 cDNA indicates that it encodes for a 1594 amino acid extracellular protein with an estimated molecular weight of 178 kDa (UniProtKB–P58397). ADAMTS12 expression was detected in a variety of tissues like cartilage, lung, kidney, liver, synovium, tendon, skeletal muscle, adipose (Liu, 2009; El Hour et al., 2010; Moncada-Pazos et al., 2012). It is also widely expressed in tumors from different origins and mainly associated with the gastrointestinal tract, suggesting that this enzyme could also participate in the development or progression of this type of tumors (Liu et al., 2006b; Moncada-Pazos et al., 2009; Wang et al., 2011; Fontanil et al., 2014; Liang et al., 2020; Rabadan et al., 2020).

**FIGURE 1:**
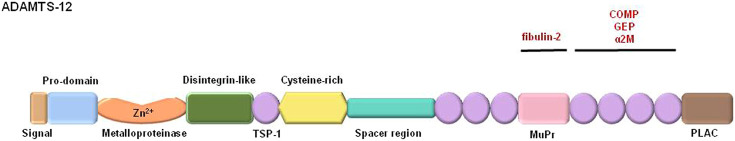
Schematic representation of human ADAMTS-12. In black are shown the main domains of the protein; TSP-1: thrombospondin-like type-1 domain; MuPr: spacer two region. In red are shown proteins known to interact with ADAMTS-12; COMP: cartilage oligomeric matrix protein; GEP: granulin epithelial precursor; α2-macroglobulin.

Several studies performed to characterize ADAMTS-12 function suggest its role as a host-protective enzyme with antitumor properties (Llamazares et al., 2007; Moncada-Pazos et al., 2009; El Hour et al., 2010; Wang et al., 2011; Rabadan et al., 2020). ADAMTS-12 also participates in other pathological situations in which a common trail of an inflammatory outburst is present such as the allergen-induced hyperresponsiveness detected in asthma or the extracellular matrix degradation observed in osteoarthritic processes (Liu, 2009; Moncada-Pazos et al., 2012; Nah et al., 2012; Paulissen et al., 2012). Furthermore, in support of previous observations, by different genomic approximations the ADAMTS-12 locus has been associated with several human pathologies like asthma, schizophrenia or predisposition to pediatric stroke (Kurz et al., 2006; Arning et al., 2012; Bespalova et al., 2012). In recent years, genome-wide and bioinformatics analysis techniques have been used with results supporting ADAMTS12 participation in those pathological processes and also in new situations with the benefit of the possibility of being used as a prognosis biomarker (Hu et al., 2020; Liang et al., 2020; Zhang et al., 2020). However, besides its participation in pathological situations much more research is needed to identify ADAMTS-12 interacting partners and its substrates in normal or pathological processes. This fact relies in the complex structure of ADAMTS-12 in which multiple domains can be identified and thus, multiple interacting regions are possible for other components of the extracellular matrix (ECM) (Figure 1).

The main processes in which ADAMTS-12 has been proposed to participate will be discussed in the following sections, focusing in the importance of those biological processes in which the associations with ADAMTS-12 have been described.

### ADAMTS-12 in Inflammation

Initial studies aimed to decipher ADAMTS-12 biological function indicate its role as a critical mediator in inflammation processes. Thus, in the human genome screening program ADAMTS12 was identified as a putative asthma associated gene (Kurz et al., 2006). Screening was made in two different populations to identify a chromosome 5-linked asthma or bronchial hyperresponsiveness locus by genotyping 89 single nucleotide polymorphisms (SNPs) in 22 genes and in two different populations (Kurz et al., 2006). Further, *in vivo* studies were made taking advantage of the availability of the *Adamts12* deficient mice and the use of different functional approaches (El Hour et al., 2010; Moncada-Pazos et al., 2012; Paulissen et al., 2012). Two different models of allergic airways disease were used to challenge the *Adamts12* deficient mice, ovalbumin (OVA) and house dust mite asthma-induced models. In absence of Adamts-12, an elevated eosinophilic inflammation, together with increased levels of mast cells and IL-33, were observed in the alveolar environment suggesting a protective role of this proteinase against this inflammatory process (Paulissen et al., 2012).

Other models of inflammation were performed in the same genetic background and compared to normal mice such as colitis, pancreatitis and endotoxic sepsis. Overall, absence of *Adamts12* in mice resulted in a more severe inflammatory phenotype. As mentioned before, a protective role of ADAMTS-12 in inflammatory processes is suggested for these three models which, at the same time, showed some common cellular and molecular patterns. Mice lacking Adamts-12 exhibited an increased tissue damage subsequent to inflammation, accompanied by high levels of pro-inflammatory cytokines like IL-6, IL-11, GCSF, S100A8, S100A9 and also hemopexin, a free heme scavenger protein known for delaying apoptosis of neutrophils (Arruda et al., 2004). Furthermore, neutrophil accumulation has been observed in all the models and in the insulted organs of *Adamts12* deficient mice which suggested a required key role of Adamts-12 in neutrophil clearance and subsequent resolution of the inflammatory process (Moncada-Pazos et al., 2012).

All together these data suggest that ADAMTS-12 has a protective role or acts as an intermediate factor in the resolution in these inflammatory processes, In addition, absence of this proteinase results in a sustain inflammatory phenotype.

### ADAMTS-12 in Orteoarthritis

Osteoarthritis (OA) and rheumatoid arthritis (RA) are believed to be the most prevalent rheumatic diseases, affecting approximately 11% (10% OA and 1% RA) of the world population (Frangos and Maret, 2020). Both, RA and OA are debilitating degenerative diseases affecting articular joints which are characterized by a deregulation in joint or bone homeostasis. Physiological balance is altered and results in the degradation of articular cartilage, alterations in the subchondral bone mass, and also it is also characterized by the presence of localized inflammatory events (Dancevic and McCulloch, 2014; Perez-Garcia et al., 2019a). In addition, this local inflammation is associated with an increase in the production of zinc-dependent matrix metalloproteases which are responsible for the degradation of main components of the extracellular matrix of the cartilaginous tissue (Kevorkian et al., 2004). MMPs and ADAMTSs are known proteases that participate in this process by cleaving some of these components; such is the case of COMP (Cartilage Oligomeric Matrix Protein) and the hyalectan aggrecan (Dickinson et al., 2003; Sandy, 2006). Among them, the association of ADAMTS-12 with arthritis and osteoarthritis has been described in several studies along with other members of the ADAMTS family such as ADAMTS-7, and the aggrecanases ADAMTS-4 and ADAMTS-5 (Lin and Liu, 2009; Lin and Liu, 2010).

ADAMTS-12 and ADAMTS-7 share a very similar structure, showing unique domains and thus forming their own subgroup within the ADAMTS family (Kelwick et al., 2015). It is suggested that both enzymes may play key roles in the onset of arthritic events through the ability of cleaving COMP, a pentameric glycoprotein that is part of the extracellular matrix and is used as an indicator of accelerated joint erosion (Liu et al., 2006a; Liu et al., 2006b; Kullich et al., 2007; Liu, 2009). The cleavage of COMP requires an interaction of the EGF-like domain of COMP with the four carboxy-end type 1-thrombospondin repeats found in both ADAMTS-7 and ADAMTS-12 (Guo et al., 2010; Lin and Liu, 2010). The recognition of these binding sites was initially observed by a yeast-two-hybrid genomic screening and subsequently demonstrated by co-immunoprecipitation techniques and *in vivo* detection (Liu et al., 2006a; Liu et al., 2006b). In fact, ADAMTS-12 participation in arthritic processes has been mainly related to its ability to degrade COMP because fragments generated following its cleavage were found in diseased cartilage, synovial fluid and in serum of patients with knee injuries, osteoarthritis and rheumatoid arthritis (Neidhart et al., 1997; Dickinson et al., 2003; Lin and Liu, 2009). Furthermore, COMP fragments observed in cartilage explants of osteoarthritis patients showed similar size to those identified from *in vitro* studies (Luan et al., 2008). Elevated levels of ADAMTS-12 in OA and RA, and ADAMTS-7 in RA were detected in the same tissues in which products generated by the cleavage of COMP were identified suggesting that COMP and these enzymes may co-localize (Liu et al., 2006a; Liu et al., 2006b; Luan et al., 2008; Lin and Liu, 2009). In 2008 Luan *et al.* demonstrated that the COMP fragments found in the cartilage of six patients with OA showed the same size (110 kDa) than those obtained by *in vitro* degradation of COMP by both proteases. In addition, in cartilage explants the expression of both enzymes increased with the expression of TNF-α and IL-1β, therefore favoring COMP cleavage. Moreover, inhibition with antibodies that block either ADAMTS-7 or ADAMTS-12 as well as the use of specific siRNAs kept COMP unprocessed in both, the explants and human chondrocytes (Luan et al., 2008). Additional finding linking ADAMTS-12 with osteoarthritis pathologies came from the identification of ADAMTS12 polymorphisms associated with RA. More specifically three SNPs: rs1364044, intron C/T; rs10461703, intron C/T; rs25754, and a missense Thr1495Ile) were identified in this pathology (Nah et al., 2012). All of it together with the fact that *ADAMTS12* is one of the genes up-regulated in OA with high levels in cartilage, synovial fluid and serum of arthritic patients supports the idea of *ADAMTS12* to be considered as a *bona fide* gene for the development of anti-RA therapies (Kevorkian et al., 2004; Liu et al., 2006b; Nah et al., 2012).

Further confirmation of ADAMTS-12 participation in osteoarthritis processes was recently demonstrated through the employment of an *in vivo* collagen-induced arthritis (CIA) model by using *Adamts12* deficient mice (Wei et al., 2018). Mice lacking Adamts-12 developed accelerated inflammatory arthritis when compared to their control littermates. Bone destruction, increase in synovitis, higher cartilage loss, and increased osteoclastogenesis were the main alterations observed in *Adamts12* deficient mice after CIA. This work also pointed out that ADAMTS-12 participation in the arthritic phenotype may be in part caused through proteolytic processing of Connective Tissue Growth factor (CTFG) by ADAMTS-12. The observed data also suggested that CTFG may be a downstream mediator of ADAMTS-12 participation in the inflammatory process (Wei et al., 2018). In this context, ADAMTS-12 is suggested to act as a factor that prevents inflammatory arthritis. In fact, absence of CTGF degradation in *Adamts12* deficient mice after IL-1β stimulation led to an increase of pro-inflammatory markers like NOS-2, IL-6 and COX-2 and a reduction of IL-10, a known anti-inflammatory molecule (Wei et al., 2018). Therefore, this data suggests again a protective role for ADAMTS-12 in arthritic events through modulation of the inflammatory landscape.

Interactions between ADAMTSs and different ECM components are of important relevance regarding the participation of these metalloproteases in the regulation of extracellular matrix homeostasis. In fact, degradation of COMP by ADAMTS-7 and ADAMTS-12 is naturally inhibited by the presence of alpha-2-macroglobulin (α2M), which is also an endogenous substrate of both proteases (Luan et al., 2008; Wan et al., 2012). ADAMTS-7 and ADAMTS-12 have also been described to interact and co-localize with Granulin-Epithelin Precursor (GEP), an autocrine growth factor expressed in different pathologies, including RA and OA (Justen et al., 2000). In turn, GEP is also able to block COMP degradation by these two enzymes (Bai et al., 2009a; Guo et al., 2010). GEP binding to either ADAMTS-7 or ADAMTS-12 matches the same domains to which COMP interaction occurs and thus can be considered as a competitive inhibitor. GEP and COMP bind to the four C-terminal TSP-I like motifs of both ADAMTSs and it does so in a dose dependent manner. GEP interaction resulted in a GEP proteolytic cleavage that releases several fragments of ∼6 kDa known as granulins that can attach to the carboxy-end of both enzymes (Davidson et al., 2004). GEP not only competes directly with COMP for ADAMTS binding but also reduces the levels of both, ADAMTS-7 and ADAMTS-12 through downregulation of TNF-α (Luan et al., 2008).

Cartilage remodeling by uncontrolled activity of chondrocytes is not the unique hallmark in the development of OA since other processes, such as synovial inflammation can also occur (Di Nicola, 2020; Rocha et al., 2021). In this sense, synovial fibroblasts (SF) can also be included as important players in joint destruction in both RA and OA with an invasive phenotype. SF, in an inflammation environment, are known to produce and secrete various members of the ADAMTS family which are able to degrade cartilage components such as aggrecan and COMP (Lefevre et al., 2015; van Nie et al., 2020). Moreover, inflammatory cytokines like IL-1β as well as fibronectin fragments can induce an increase of ADAMTS-12 expression, both at the mRNA and protein levels, in SF from OA patients when compared with healthy synovial fibroblasts and therefore increase the degradation of COMP. Something similar occurs with ADAMTS-7 with the difference that in this case it occurred in both types of fibroblasts (Perez-Garcia et al., 2016).

Mechanism by which IL-1β mediates ADAMTS-7 and ADAMTS-12 expression in SF was recently described and involves two different pathways, Runx2 and Wnt/*β*-catenin. Presence of specific inhibitors for both pathways in SF co-cultured with cartilage explants from patients with OA were used to demonstrate that ADAMTS-7 expression depends on the Wnt/*β*-catenin pathway, whereas ADAMTS-12 depends on the ERK pathway and ultimately in the activation of Runx2. It was also shown that IL-1 *β* can induce expression of ADAMTS-7 and ADAMTS-12 which led to COMP degradation. However, while IL-1β can induce both ADAMTSs, the presence of fibronectin fragments was only able to induce ADAMTS-12 expression and the consequent COMP degradation (Perez-Garcia et al., 2019b).

In summary, involvement of ADAMTS-12 in arthritic processes has been demonstrated by its ability of degrading essential components such as COMP. Production of inflammatory mediators (for example IL-1β) induces ADAMTS-12 expression which through COMP cleavage releases other component of the extracellular matrix with proinflammatory effects. As a result, all these molecular interactions generate a positive feedback loop that increases joint damage which is a characteristic observed in osteoarthritic processes (Liu et al., 2006b; Perez-Garcia et al., 2016; Perez-Garcia et al., 2019b). In addition, ADAMTS-12 seems to be also responsible for limiting inflammatory arthritis by modulating pro- and anti-inflammatory molecules. Degradation of other ECM molecules like CTFG can be considered part of an important mechanism for the resolution of the arthritic inflammatory phenotype.

### ADAMTS-12 in Chondrogenesis

ADAMTS-12 plays an important role in the regulation of chondrogenesis and cartilage development. Levels of ADAMTS-12 have been detected strongly upregulated during chondrogenesis and also in proliferating and hypertrophic chondrocytes (Jin et al., 2007; Bai et al., 2009a; Bai et al., 2009b; Jahangir et al., 2020). ADAMTS-12 role in chondrogenesis is related to an important chondrogenic regulator, Parathyroid Hormone-Related Peptide (PTHrP) (Rajagopal et al., 2020). A feedback loop beween PTHrP and ADAMTS-12 has been observed in terms that ADAMTS-12 induces PTHrP and ADAMTS-12 is barely detectable in PTHrP -/- growth plate chondrocytes since PTHrP, at the same time induces ADAMTS-12 expression. The effects caused by these two molecules result in the inhibition of chondrocyte differentiation through the inhibition of genes such as type II collagen (Col II) or Sox9, markers of chondrocyte differentiation (Bai et al., 2009b).

On the other hand, genetic studies in a family of patients with autosomal-dominant Brachydactyly Type E disorder had led to the identification of a (8; 12) (q13; p11.2) translocation that affected the PTHLH gene which encodes for PTHrP (Maass et al., 2010). Since this translocation affects PTHrP locus, it also has consequences on its downstream targets, ADAMTS-7 and ADAMTS-12, therefore affecting normal chondrogenic differentiation.

Another molecule that regulates the activity of ADAMTS-12 in chondrogenesis is the transcription factor c-Maf, a member of the Maf family of basic ZIP transcription factors (bZIP). Among other functions, c-Maf is known for its involvement in chondrogenesis (Hong et al., 2011; Hong et al., 2013). Thus, expression of c-Maf is maximal in hypertrophic chondrocytes during embryonic development and postnatal growth. On the other hand, ADAMTS-12 expression increases during *in vitro* chondrogenesis of human mesenchymal stem cells as well as during mouse embryonic limb development (Bai et al., 2009b; Hong et al., 2011), A “Maf recognition element” (MARE) has been identified in the ADAMTS12 proximal promoter where, through the use of luciferase reporter assays, c-Maf binding has been demonstrated to induce ADAMTS-12 expression. Therefore, c-Maf effects on differentiation and hypertrophy of chondrocytes might be in part through up regulation of ADAMTS-12 (Hong et al., 2013).

### ADAMTS-12 in Degenerative Intervertebral Disc

Between each vertebral body of the spine are pads of fibrocartilage-based structures that provide support, flexibility, and a better load distribution known as intervertebral discs. Each of them also contains a soft tissue within the interior which is known as nucleus pulposus (Donnally et al., 2021). As happens in arthritic processes and other inflammatory disorders, the intervertebral discs can be subjected to degradation by molecules capable of processing extracellular matrix components as is the case of MMPs and ADAMTSs (Le Maitre et al., 2007). In this regard, high levels of ADAMTS-7 and ADAMTS-12 have been observed in nuclei pulposus extracted from rats in which disc degeneration was generated through the use of an external compression device (Yu and Zhu, 2012). These results were accompanied by an increase in COMP fragments (Liu et al., 2006b; Yu and Zhu, 2012). In addition, levels of both proteases are increased in endplate cells isolated from patients with IVD suggesting their participation in this pathological process through degradation of the ECM components (Zhang et al., 2012).

All of these data would indicate that ADAMTS-7 and ADAMTS-12 contribute to intervertebral disc degeneration through a molecular mechanism similar to that described for the osteoarthritic disorders (Yu and Zhu, 2012; Zhang et al., 2012).

### ADAMTS-12 in Ossification and Tendon Degeneration

Presence of higher levels of ADAMTS-7 and ADAMTS-12 at mRNA and protein levels in degenerative IVD when compared with normal IVD seems to be associated with low levels of Col II, Sox9 and Col X, known chondrogenesis marker genes (Zhang et al., 2012). ADAMTS-7 and ADAMTS-12 are associated to cartilage destruction and endochondral ossification (Lin and Liu, 2009). *Adamts7* and *Adamts12* deficient mice are viable with no apparent phenotype. *Adamts7* knockout was initially used to validate the association of Adamts-7 with atherogenesis while *Adamts12* knockout served as a model to describe Adamts-12 antitumoral function (El Hour et al., 2010; Bauer et al., 2015). Both proteases share an important structural homology and have shown mutual compensation in hindlimb tendons, with upregulation of one of them when the other one is absent (Mead et al., 2018). Double knockout of both ADAMTSs has been generated to prove their participation in skeletal development and more specifically, in heterotopic ossification (HO) of tendons and ligaments (Mead et al., 2018). Furthermore, these mice developed normally, with no skeletal anomalies till the age of 4 months when HO was identified within the quadriceps tendon, Achilles tendon and menisci (Mead et al., 2018). Extension of the study to human pathologies showed that reduction of ADAMTS-7 and ADAMTS-12 immunostaining was evident in human degenerative biceps tendons although only ADAMTS7 mRNA was reduced significantly (Mead et al., 2018).

Participation of ADAMTS-12 and ADAMTS-7 in ECM remodeling in musculoskeletal tissues is important for homeostasis maintenance. The above studies indicate that both proteases are basally expressed in bone, cartilage, synovium, ligament and tendons and thus being implicated in normal turnover of these tissues. Alterations on their expression could lead to the development of different pathologies like arthritis, IVD degeneration and ossification. Furthermore, the structural similarity of ADAMTS-7 and ADAMTS-12 can generate compensatory mechanisms between these proteases in those body compartments in which both are normally expressed.

### ADAMTS-12 in Neurological Disorders

In 2012, Bespalova et al. (Bespalova et al., 2012) identified a schizophrenia susceptibility locus on chromosome 5p13. This region contains the ADAMTS12 gene and to study its potential involvement in the disease, a mutation analysis was performed in Puerto Rican patients of Spanish descent. That analysis revealed that one intronic variant and two SNP haplotypes were closely linked to the susceptibility of developing schizophrenia. Previously, sequence variants in the ADAMTS12 gene had also been associated to bipolar disorder and narcolepsy using genome-wide association studies (GWAS) in Japanese population (Hattori et al., 2009; Koike et al., 2009). GWAS technique has also allowed to include ADAMTS12 gene as functionally linked to Alzheimer´s disease (Wang et al., 2015). Taking together, those studies showed clear evidence that genetic variations of the ADAMTS12 gene could underlie mental health disorders.

Also in 2012, a study was published highlighting the association between the ADAMTS12 gene and pediatric stroke using GWAS (Arning et al., 2012). Further evidence that ADAMTS12 could display a role in pediatric stroke was obtained through the sequencing and fine mapping of ADAMTS12 gene variants (Witten et al., 2020). Similar type of analysis was performed to determine that an ADAMTS-12 variant could offer a protective effect against cerebral aneurysm (Arning et al., 2016). These results suggest that ADAMTS12 could be considered a very promising factor to analyse in relation to its capacity to associate to other ECM components of the CNS. Moreover, ADAMTS-12 might also potentially induce modifications in ECM components through its catalytic activity. In consequence, an altered ADAMTS-12 metalloprotease could lead to a disrupted ECM. These studies also open the possibility of ADAMTS-12 could be a potential diagnostic and prognostic biomarker in cerebrovascular diseases. However, it would be necessary to explore the functional mechanisms associated to ADAMTS-12 activities in neurological system.

In order to shed light about the functional role of ADAMTS12 in the central nervous system, we recently studied its expression in different areas of mouse brain at different developmental stages (Fontanil et al., 2019). Expression was detected at different levels in embryonic stages, but was very low or undetected in the postnatal or adult stages analyzed. As a potential substrate of ADAMTS-12 in brain, neurocan expression was also evaluated with the finding that this hyalectan displayed a similar expression pattern as that shown by the metalloprotease. Gene expression analysis indicates that both, neurocan and ADAMTS12, can be detected in the olfactory bulb in the brain of adult mice. *Adamts12* knockout mice develop normally with no apparent behavior deficiencies or CNS alterations. However, the absence of Adamts-12 results in neurocan accumulation in specific areas of the CNS such as olfactory bulb, hypothalamus and spinal ganglia of new born mouse (Fontanil et al., 2019). This accumulation potentially excludes neurocan processing by other proteases. Neurocan cleavage not only by ADAMTS-12 but also by other ADAMTS family members could be crucial for normal maintenance of brain function (Tauchi et al., 2012; Gottschall and Howell, 2015; Fontanil et al., 2021). In this regard, neurocan has been related with important functions in brain development. For instance, it has been recently reported that neurocan contributes to the perineuronal nets formation during postnatal development in mouse (Schmidt et al., 2020). Moreover, neurocan levels increase during traumatic brain injury (Asher et al., 2000), which could contributed to generate a three-dimensional net together other ECM components for cells in order to repair the damage (Fontanil et al., 2019). It is also noteworthy that gene encoding neurocan, NCAN, has been associated with mental health diseases (Avram et al., 2014) including schizophrenia and bipolar disorder (Muhleisen et al., 2012; Oruc et al., 2012; Wang et al., 2016) or related with the etiology of mania (Miro et al., 2012). Different studies have connected the rs1064395 SNP within NCAN gene with those disorders (Raum et al., 2015; Wang et al., 2016). It has been recently shown that the NCAN rs1064395 A allele is linked to lower hippocampus-dependent memory function (Assmann et al., 2020). Furthermore, that SNP influences the expression of the neighboring HAPLN4 gene (Assmann et al., 2020). This gene codes for the ECM link protein Hapln4/Bral2, which show a strong expression in human cortex (Bekku et al., 2003; Spicer et al., 2003). These studies point to the strong influence of this genetic variant of the NCAN gene on the ECM composition, highlighting the influence of neurocan in both normal and pathological conditions of the CNS. In addition, a growing body of evidence suggests that ADAMTS-12 could influence brain function through different mechanisms, including its functional relationship with neurocan.

### ADAMTS-12 in Cancer

ADAMTS-12, like many other members of the ADAMTS family, may have a pro- or anti-tumor role in various types of cancers. Initially, ADAMTS-12 was described as a tumor suppressor protein, as the exogenous expression of ADAMTS-12 in Madin-Darby Canine Kidney (MDCK) cells prevents the phenotypic changes associated with renal carcinogenesis caused by the growth factor of hepatocytes (HGF). This effect is due to the thrombospondin motifs that this enzyme exhibits at the C-terminal end, and which block the Ras-MAPK signalling pathway (Llamazares et al., 2007). MDCK cells expressing a truncated form of ADAMTS-12 lacking the TSP-1 repeats also showed tumorigenic effects compatible with those previously described. ADAMTS-12 overexpression in A549 lung adenocarcinoma cells inhibits subcutaneous tumor formation in immunodeficient SCID mice in comparison with animals injected with A549 parental cells. In addition, ADAMTS12 gene is epigenetically silenced by hypermethylation of its promoter in tumors of different sources, which reinforced the hypothesis of ADAMTS12 as a tumor suppressor (Moncada-Pazos et al., 2009). Furthermore, in colon tumors there is a dual regulation of ADAMTS-12 expression: first, an epigenetic inactivation in colon cancer cells due to high levels of methylation of ADAMTS12 promoter. And second, an overexpression of ADAMTS-12 by the fibroblasts surrounding neoplastic cells was detected, which suggest a protective stromal response to reduce tumor progression. Other studies support the protective role of ADAMTS-12 in colorectal cancer (Wang et al., 2011; Zheng et al., 2019). However, it has also been described that ADAMTS-12 can promote the migration and proliferative properties of HCT116 colorectal carcinoma cells by activating the Wnt/*β*-catenin signaling pathway (Li et al., 2020a). On the other hand, the phenotypic analysis of the *Adamts12* deficient mouse indicated that this protein plays a protective role against angiogenesis and tumor growth. In fact, mice lacking an active Adamts-12 showed high levels of vascularization and tumor invasion after malignant keratinocyte transplantation (El Hour et al., 2010). These mice also showed a greater number of lung tumors when compared with their normal littermates after administration of the carcinogen urethane. In addition, *in vitro* studies using M-38 lung carcinoma cells revealed an increase in their proliferative and invasive potential after being depleted of ADAMTS-12 using RNA interference techniques. Protective role of ADAMTS-12 in lung cancer was also supported through the employment of bioinformatic approaches. This analysis would indicate that patients with lung adenocarcinoma in which truncating mutations in the ADAMTS12 gene were detected, would have a worse prognosis than those with the unaltered gene (Rabadan et al., 2020).

A protective role of ADAMTS-12 has also been found in breast cancer through its association with fibulin-2 (Fontanil et al., 2014). In this case, fibulin-2/ADAMTS-12 interaction was demonstrated after yeast-two-hybrid screening and inmunoprecipitation studies. ADAMTS-12 exogenously expressed in breast cancer cells increases the formation of subcutaneous tumors in immunodeficient SCID mice, and the capacity of migration, invasion, and mammosphere formation of these cancer cells, which can be associated to a pro-tumoral effect. However, presence of fibulin-2 and ADAMTS-12 is able to block those properties suggesting a protective role induced by that interaction (Fontanil et al., 2014). In fact, fibulin-2 can be degraded by ADAMTS-4 and ADAMTS-5 under the same experimental conditions, which results in an increase of the tumoral properties of breast cancer cells (Fontanil et al., 2017). The interaction of ADAMTS-12 with fibulin-2 results in a blockade of the proteolytic degradation of fibulin-2 by ADAMTS4 and ADAMTS-5 and therefore, inhibition of the tumoral properties of breast cancer cells (Fontanil et al., 2017).

In a stark contrast, ADAMTS-12 has also been associated to protumoral effects. Thus, it has been shown that the overexpression of ADAMTS-12 in human trophoblastic cells enhances their invasive phenotype through regulating the expression and function of the integrin α_v_β_3_ (Beristain et al., 2011). In addition, ADAMTS-12 plays a pro-tumoral role in breast cancer, by increasing the formation of subcutaneous tumors in immunodeficient SCID mice, and the capacity for migration, invasion, and mammosphere formation in breast cancer tumor cells. However, when ADAMTS-12 interacts with fibulin-2, these effects are reversed, promoting an antitumor role (Fontanil et al., 2014). Other studies seem to suggest the tumorigenic function of this protein since the gene encoding for ADAMTS12 has been identified as one of the genes overexpressed in ovarian cancer patients with intestinal metastases (Mariani et al., 2019), in patients with metastatic renal carcinoma (Ho et al., 2017), gastric carcinoma with poor prognosis (Liang et al., 2020) and esophageal squamous cell carcinoma (Li et al., 2020b).

With the previous data on mind, analysis and correlation studies are of key importance to decipher the precise role of ADAMTS-12 in cancer development. In fact, ADAMTS-12 seems to be preferentially associated with cancer associated fibroblasts (CAFs) and immune cells (macrophages) in colorectal cancer (Moncada-Pazos et al., 2009; Wang et al., 2011). ADAMTS-12 has been specifically detected in activated fibroblasts in the proximity of colon cancer cells. Furthermore, co-cultured fibroblasts are able to induce colon cancer cells death by apoptotic mechanisms (Moncada-Pazos et al., 2009). In the urethane-induced lung cancer model, ADAMTS-12 is present in cells surrounding the highly proliferative cells within the tumor (Rabadan et al., 2020). In breast cancer, ADAMTS-12 expression is localized preferentially surrounding the tumoral tissue and high expression is correlated with good prognosis of these patients (Fontanil et al., 2014). On the other hand, expression analysis in gastric cancer has revealed high levels of ADAMTS-12 in tumoral samples when compared to adjacent tissue and a correlation with poor prognosis (Liang et al., 2020). In this sense, the availability of gene expression databanks can be a great tool to analyze gene participation in cancer. However, care must be taken in order to define gene groups when analyzing complex samples from tumors in which types of cells from different origin can be found (fibroblasts, cancer cells, endothelial, muscle cells and immune cells among others). In addition, ADAMTS-12 participation in the resolution of inflammatory processes should also be taking into account in local cancer development. Inflammatory molecules and the immune response have been described to be drivers and to potentiate cancer development (Suarez-Carmona et al., 2017; Gupta et al., 2018). Pro-inflammatory cytokines are also potent chemoattractants for immune cells that, consequently, increase the local inflammatory environment. ADAMTS-12 participation in modulation and resolution of inflammatory processes might as well be part of its antitumoral effect. In fact, ADAMTS-12 can reduce levels of inflammatory molecules by degradation of CTFG and, at the same time it is also able to induce neutrophil clearance by inducing cell apoptosis (Moncada-Pazos et al., 2012; Wei et al., 2018).

In conclusion, ADAMTS-12 can be related to act as a tumoral suppressor and also to act as a protumoral agent. In this sense, ADAMTS-12 function may depend on several factors, type of cells in which it is expressed (cancer cells, inmune or stromal cells), the appearance of ADAMTS-12 mutants (point mutations, truncated forms, protein processing) as well as the interactions with other components of the ECM.

### ADAMTS-12 in Fertility Disorders

The initial phenotypic analysis of *Adamts12* deficient mice showed no alterations in reproduction, with normal gestation periods and fertility. However, the expression of *Adamts12* in wild type mice was detected in tissues such as the mammary glands, uterus and ovary, but not in testes (El Hour et al., 2010). In addition, ADAMTS-12 plays a role in angiogenesis and inflammation, which may imply that this protein can be involved in processes such as the formation of the placenta and problems associated with pregnancy as is the case of pre-eclampsia. Moreover, ADAMTS-12 affects the invasion of trophoblastic cells and their adhesion to components of the extracellular matrix by regulating the expression of the integrin α_v_β_3_ (Beristain et al., 2011). In fact, high levels of ADAMTS-12 are detected in placenta during the first three months of pregnancy, which highlights the important role that this protein plays in the implantation of this organ. Regarding the role of ADAMTS-12 in pre-eclampsia, data are controversial, as a decrease in ADAMTS-12 levels have been detected in women with pre-eclampsia (Eda Gokdemir et al., 2016). However, other studies found an increase in ADAMTS-12 levels in placenta (but not in maternal blood or umbilical cord) in patients with pre-eclampsia (Namli Kalem et al., 2018). Finally, other studies did not detect differences in these groups (Daglar et al., 2016; Kirbas et al., 2016; Oztas et al., 2016) but a decrease in levels of ADAMTS-12 in placenta during pregnancy-associated cholestasis (Oztas et al., 2016). Recently, it has also been reported that women with spontaneous preterm birth present hypomethylation of ADAMTS12 promotor in the placenta tissue (Mani et al., 2019).

### Perspectives in ADAMTS-12 Functional Biology

**FIGURE 2:**
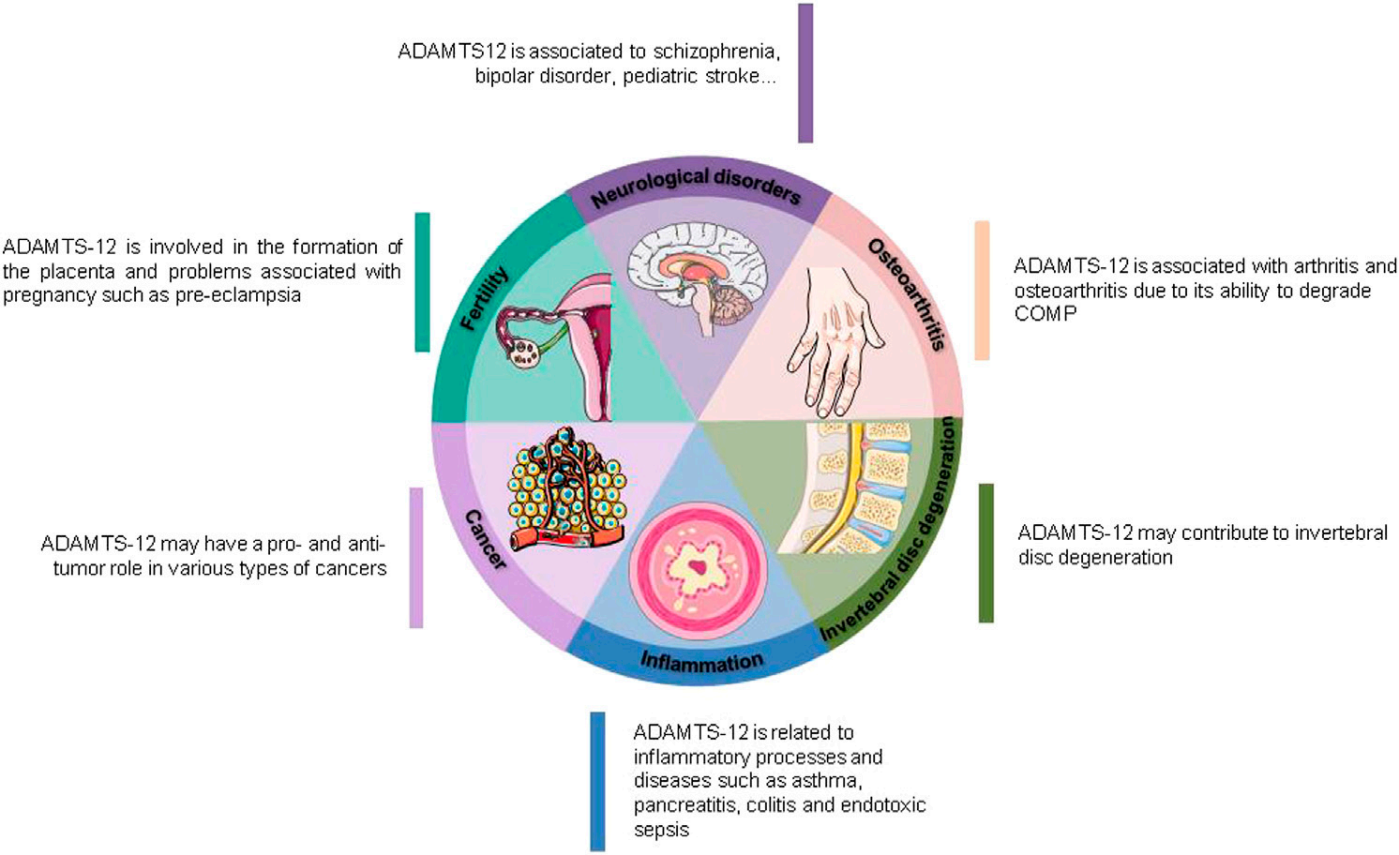
ADAMTS-12 biological implications.

During the past two decades several studies have associated ADAMTS-12 with different pathologies as well as being part of physiological processes (Figure 2). ADAMTS-12 has been initially identified as a member of the wide family of Zn^2+^-metalloproteases. However, ADAMTS-12 needs to be considered much more than a simple proteinase due to its complex structure that contains multiple different domains able to interact with other components of the ECM. These interactions not only could be important for the proteolytic activity of the enzyme but can also sequester different factors, bring to proximity components of the extracellular matrix or trigger cellular responses. For instance, it is known that ADAMTS-12 interacts with other ECM components like fibulin-2, CTFG, COMP, GEP, α2M and can also interact with growth factors such as VEGF (Liu et al., 2006b; Llamazares et al., 2007; Luan et al., 2008; Wan et al., 2012; Fontanil et al., 2014; Wei et al., 2018). In addition, ADAMTS-12 is known to be processed which generates two fragments, one with the proteolytic domain (amino-terminal end) and the other one containing different anchoring domains (mainly TSP-1 repeats) (carboxy-terminal end) which, in turn, can generate the appearance of different activities (Llamazares et al., 2007; Beristain et al., 2011; Wei et al., 2018). All these facts could be relevant to ADAMTS-12 biological function since it is generally accepted that C-terminal domains of metalloproteinases are often important for determining substrate specificity (Martel-Pelletier et al., 2001).

ADAMTS-12 participates in different processes related to an inflammatory response such as asthma, colitis, sepsis, pancreatitis and osteoarthritis. In this sense, mechanisms about ADAMTS-12 participation and regulation of these inflammatory events are known to be through interaction and degradation of different components of the ECM. It is interesting to note that this enzyme is able to cleave key proteins of cartilage like COMP and, at the same time, degrade other components like CTGF which seems to be required for reducing the inflammatory response (Liu et al., 2006b; Wei et al., 2018). In addition, ADAMTS-12 may have other anti-inflammatory actions by blocking the appearance of elevated levels of pro-inflammatory molecules as well as a role in neutrophil clearance (Moncada-Pazos et al., 2012). Therefore, ADAMTS-12 can be considered an important player in mediating and resolving the inflammatory response and, its absence may result in pathologies known to be due in part to a not well resolved inflammatory process like can be the case of schizophrenia (Asher et al., 2000; Fontanil et al., 2019).

In summary, ADAMTS-12 can be a key player in the control and regulation of different biological processes, both normal and pathological. It is clearly associated with inflammatory processes but also seems to have an important role in cancer, neurological disorders as well as being related with fertility disorders. Further studies on patients with infrequent anomalies as well as those that have been treated with radioisotopes have found that ADAMTS-12 can also be part of the biological mechanism of Freiberg-s infraction, brachydactyly A1B as well as salivary gland damage (Koca et al., 2013; Mysliwiec et al., 2015; Sadic et al., 2016).

As mention in this review, ADAMTS-12 has a complex architecture which can support different activities and hence, be associated to different physiological a pathological processes. The availability of mice deficient in this protease is a useful tool in order to demonstrate its participation in those processes through the use of different experimental models. Furthermore, bioinformatics applications are also being widely used to make associations of ADAMTS-12 with several human diseases. However, more bench work is needed to solve ADAMTS-12 biological function, its interactions with other components of the ECM, the existence of specific substrates and the implications of the different parts of the molecule in regulating those functions. It would be also crucial to identify new interacting partners of this complex metalloprotease within the ECM. This would open the possibility to clarify controversial aspects about the functional relevance of ADAMTS-12.
